# The Efficiency of Poultry Farms: A Dynamic Analysis Based on a Stochastic Frontier Approach and Panel Data

**DOI:** 10.3390/ani15192806

**Published:** 2025-09-26

**Authors:** Maria Bonaventura Forleo, Paola Di Renzo, Luca Romagnoli, Vincenzo Giaccio, Alfonso Scardera

**Affiliations:** 1 Department of Economics, University of Molise, Via De Sanctis, 86100 Campobasso, Italy; paola.direnzo@unimol.it (P.D.R.); luca.romagnoli@unimol.it (L.R.); giaccio@unimol.it (V.G.); 2 Council for Agricultural Research and Economics (CREA), Via G. Vico, 4, 86100 Campobasso, Italy; alfonso.scardera@crea.gov.it

**Keywords:** poultry sector, financial items, technical inefficiency, fixed effects, panel data, FADN, Italy, SFA, COVID-19

## Abstract

EU production is important for global poultry markets and is concentrated in a few countries, including Italy. The aim of this study is to investigate the technical efficiency of Italian poultry farms in 2019–2022, characterized by the COVID-19 pandemic and avian influenza, which occurred almost simultaneously and presented poultry farms with important economic challenges. In particular, this study aims to observe how efficiently poultry farms utilized their inputs with regards to controllable or managerial factors and exogenous shocks and factors beyond the firm’s control. Data was retrieved from the RICA database, the Italian section of the EU Farm Accountancy Data Network. After a descriptive analysis, a stochastic frontier model was applied to the panel data to estimate production frontier and firm-specific inefficiency factors. Results reveal the relevance of certain cost categories (feed, water, fuel, and electricity) and their increase over the observed period. Current and capital costs have positive and significant impacts on the value of production. As regards the determinants of technical efficiency, a greater endowment of some inputs (labor and farm area) and the sizes of farms in terms of livestock units are correlated with an improvement in the technical efficiency of farms.

## Simple Summary

Global poultry production has seen a dramatic rise over the last 50 years and is expected to increase further to satisfy growing demand. Understanding how efficiently firms utilize their inputs to generate increasing outputs is crucial and takes on greater importance in periods of market turbulence that can affect the performances of firms, such as the recent years, characterized by avian influenza outbreaks and the COVID-19 pandemic. The findings of this study indicate that the efficiency of Italian poultry farms during this period was attributable to the management of farms’ assets, the availability of resources, and some characteristics of farms. Improving the management of capital endowment and current costs is essential for supporting farms’ economic performance and resilience, as well as for ensuring product quality and safety and animal welfare.

## 1. Introduction

Poultry production plays a significant role in the livestock sector. The global production of poultry meat has seen a dramatic rise over the last 50 years, multiplying significantly since 1961. Poultry is the livestock sector with the strongest relative change over the entire period and compared to the first two decades of the new millennium [1]; from 2020 to 2023, the increase continued (+7%), and the production value reached 144.2 million tons, but it was surpassed by that of pig meat production (15%). As regards prospects, according to an OECD-FAO report [2], global poultry production and demand is expected to increase by 2035, despite increases in energy and feed costs, because poultry products are cheaper alternatives to other animal proteins. The EU production counts for 9.3% of the global total of poultry meat and is highly concentrated in a few countries [3], in descending order, Poland (20.6%), Spain (12.9%), Germany (11.7%), France (11.5%), and Italy (10%).

Since the beginning of the decade, and especially between 2019 and 2022, several exogenous shocks, such as the pandemic and avian influenza events, have hit the sector at a global level and raised the cost of raw materials, mainly energy and feed. The European poultry sector suffered a contraction in production, mainly because of avian influenza (which mostly concerned the northern countries), but it recovered in recent years, on both the demand and the supply sides. Moderate growth in production was recorded in the most important European countries in the poultry sector, such as Poland, Spain, and Germany.

As regards Italy, the sector represents 7.8% of the value of the national agricultural production and 3.9% of the agro-industrial turnover, and it constitutes a significant part of the livestock market [4]. From 2019 to 2021, the Italian sector experienced declines in production volumes because of COVID-19 and avian influenza, and it was unable to satisfy domestic demand. Chicken prices at source found new momentum in 2022, supported by a sharp reduction in supply and increases in the costs of raw materials, which had a significant impact on production costs and net profit, most of all in the first period of COVID-19 lockdown [5,6].

Considering the above trends, understanding how efficiently firms utilize their inputs to generate output is crucial in economic analysis and takes on greater importance in periods characterized by turbulence that can affect the performances of firms.

The concept of efficiency pertains to the capability with which farms utilize their inputs to achieve optimal output. This process entails the minimization of waste and the maximization of productivity, given the available resources and technological capabilities [7]. In other words, it is considered that a farm is technically efficient when it operates at maximum output capacity, considering the inputs and technology utilized. Conversely, this denotes the process of attaining the desired level of production with the minimum possible input [8]. That means lower costs and higher profits.

The analysis of technical efficiency implies an assessment of the extent to which a farm converts inputs into outputs and the degree to which those outputs translate into financial performance. This analysis can reveal areas where a farm excels or struggles, thus guiding decisions to improve both technical and financial results. It is hypothesized that large farms may enhance technical efficiency by leveraging economies of scale [9], thereby facilitating the adoption of novel technologies and optimal management practices [10,11], including planning and organizational strategies [12]. Regarding the inputs employed in production processes, the efficient utilization of fertilizers, energy, water, and labor is imperative [13]. Poultry production systems should be optimized to enhance their resource efficiency by developing necessary infrastructure across all stages of production; digital technologies may support maximizing production efficiency, most of all in contexts of growing frequency of crises, such as the recent COVID-19 epidemic [14].

As regards the approaches used to investigate technical efficiency, researchers have applied both stochastic frontier analysis [15,16,17,18,19,20,21,22,23] and data envelopment analysis [24,25]. The measurement of technical efficiency is typically accomplished through the utilization of a “frontier” of best-practice farms as a benchmark. As [17] stated, the choice between the two approaches is dependent on the research objectives, the nature of the farms involved, the available data, and the sample size.

The financial implications of poultry production have been shown to have considerable effects on the overall profitability of poultry farming [26]. This has been particularly pronounced during the period of the global pandemic of COVID-19 (SARS-CoV-2). As [27] demonstrated, production costs, market prices, and efficient resource management play crucial roles in determining the profitability of a poultry operation.

This study provides an original contribution to the literature on poultry sector efficiency by addressing the case of Italy, a case study whose relevance lies in the paucity of literature on the topic [28]. Furthermore, as reported by Jones [29], the poultry disease literature has a primarily epidemiological focus, with very few publications providing estimates of the financial impacts of diseases.

In particular, the aims of this study are to investigate the technical efficiency of Italian poultry farms along the time span from 2019 to 2022, and to observe how efficiently they utilized their inputs with regards to controllable or managerial factors and exogenous shocks and factors beyond the firm’s control. The attention paid to the Italian case is motivated by two types of factors, mentioned above: the importance that the country has to the European poultry sector; and the importance that the poultry sector has to the Italian livestock and agricultural economy. The temporal reference of the analyses to 2019–2022 places emphasis on a period characterized by events, such as the pandemic and avian influenza, which occurred almost simultaneously and that presented poultry farms with important economic challenges. These years were characterized by significant market turbulence, including price volatility, rising input costs, and declining production volumes, which may have impacted companies’ economic and financial performances.

Aware that these characteristics make the dynamics of the time span from 2019 to 2022 significantly different from those preceding the external shocks and those occurring in more recent years, and that the observed period is short enough to capture long-term effects, the choice to focus the analysis on these years was intended to emphasize the effects that market turbulence had on companies’ short-term performances and their managerial efficiency.

After a descriptive analysis of the most relevant economic variables observed in the period, and of their dynamic, a stochastic frontier model applied to panel data of Italian poultry farms allows us to estimate the production frontier and the firm-specific factors of inefficiency over the observed time span.

## 2. Materials and Methods

### 2.1. Materials

The data for the analyses were retrieved from the RICA database, the Italian section of the EU Farm Accountancy Data Network (FADN), managed by the CREA, the National Council for Agricultural Research and Economics. The RICA datasets collect detailed technical, economic, and structural information on Italian agricultural enterprises.

The selection of farms was based on two fundamental criteria. The first criterion concerns the Technical–Economic Orientation (TEO): in accordance with the FADN classification of agricultural activities, only farms whose main activity falls under poultry farming were included. The second requirement relates to data completeness during the entire observation period, as well as consistency, meaning that the selected farms present a complete and coherent dataset for all recorded economic and structural variables.

The analyzed sample includes 122 Italian poultry farms, observed over the period from 2019 to 2022, for a total of 488 observations in a balanced panel format. For these farms, balance sheet items and some structural data were considered.

The whole dataset includes several variables concerning the structural characteristics of the farms and all items included in their financial statements. The construction of the dataset required careful selection and organization of variables that are essential for specifying both the production frontier and the inefficiency model.

A preliminary analysis of the literature was useful for selecting the main variables adopted in the studies.

The literature draws attention to the considerable number of variables that must be considered when assessing a farm’s technical efficiency. In the context of livestock farming, the measurement of technical efficiency is predicated on the consideration of key inputs, including feed, labor, and land use, with a view to producing outputs.

Revenues, capital costs, and current costs are the items most used to evaluate the financial results of the technical performance of an agricultural or livestock farm. Standardized total gross revenue is widely regarded as the total return on an investment prior to any deductions and is frequently used to evaluate the genuine financial implications of an investment, as well as to facilitate a comparative analysis between potential investments. This variable is preferable to net income, as many poultry farms have contracts that could exclude certain costs, such as veterinary fees, external services, etc., which are borne by the chicken owner [30].

The costs associated with feeding, energy, veterinary drugs, labor, veterinary services, infrastructure, and equipment are all significant factors in determining the technical efficiency and profitability of a livestock farm [17,20,25]. This is of relevance to the poultry sector, where a significant proportion of these costs could be governed by contracts [17].

The livestock unit is a variable often considered in the literature to measure the technical efficiency of a livestock farm and evaluate productivity [31]. It is a standardized measure that facilitates the comparison and aggregation of diverse livestock types. Furthermore, it is a variable that is well-suited to the two main categories of poultry farms (layers, broilers) to determine overall poultry density, feed management strategies, and feed costs [32].

The utilized agricultural area (UAA) is another factor considered when measuring the technical efficiency of a poultry farm. The land can be used to grow crops for poultry feed and to manage poultry waste, which generates valuable soil fertilizer [33]. This affects both large and small farms, including contracted and extensive poultry farms [34]. As feed is one of the main production costs for poultry farms, producing it in-house can provide a competitive advantage [35,36].

Based on the above literature, the main variables included in the econometric analysis are described below.

The stochastic frontier model has ten parameters, with four variables associated with the frontier production function, four explanatory variables influencing the level of technical efficiency, and the parameters related to the distribution of random errors.

As regards the frontier production function, the model included one output variable and three input variables, as follows:

The Value of Production (VoP, EUR) captures the total value of the revenue from livestock production of the farm, expressed in euros. It represents the output of the production function, reflecting the farm’s income-generating capacity from poultry activities.

Current Costs (CurCs, EUR) include all operating expenses incurred in the current year, such as feed, veterinary drugs, water, energy, routine maintenance, and external services. They reflect the short-term intensity of input use.

External Service Costs (ESCs, EUR) include expenses related to ancillary activities, health and veterinary services, and insurance, provided by third parties.

Capital Costs (CapCs, EUR) comprise depreciation of fixed assets and other capitalized expenditures, representing long-term investment in assets.

As regards the determinants of technical efficiency that may affect the performances of farms, this study adopts the following variables:

Livestock Units (LivUs) is a dimensional variable that quantifies the farm’s livestock capital by converting distinct categories of poultry (e.g., laying hens, broilers) into a standard unit. It serves as a proxy for the scale of poultry production.

Utilized Agricultural Area (UAA, ha) indicates the land area (in hectares) used for agricultural activities. While poultry farming is typically intensive and land-light, UAA may capture integration with on-farm feed production or management of manure.

Labor Units (LabUs) is a variable that measures total labor input, whether hired or family-based, expressed in standard annual labor units. It reflects the overall labor intensity of the production process.

TEO_Poultry (TEO_P) is a categorical variable expressing the principal Technical–Economic Orientation in poultry farming. The orientation was subdivided into two categories: TEO_PMeat, for the firms having the highest VoP share in meat production; and TEO_POther, for firms with principal orientation towards eggs or mixed meat–egg production.

### 2.2. Methods

As previously argued, understanding how efficiently firms utilize their inputs to generate outputs is crucial in economic analysis. Traditionally, production functions represent the maximum possible relationships between inputs and outputs. However, in practice, firms rarely operate at their full potential capacity.

Two main methods are used in the literature for the estimation of the efficiency of livestock farming [28]: stochastic frontier analysis (SFA) and data envelopment analysis (DEA).

Stochastic frontier analysis, a powerful econometric methodology introduced by Aigner et al. [37] and developed by Battese and Coelli [38], measures and explains a firm’s deviations from its potential production frontier by focusing on the nature of such deviations.

Data envelopment analysis is a non-parametric and deterministic method that attributes every deviation from the estimated efficiency frontier entirely to technical inefficiency. According to DEA, if a firm fails to achieve the maximum possible output from its inputs, this is solely due to intrinsic managerial or operational shortcomings. These models do not account for the influences of random external factors or measurement errors in the observed data.

In contrast, SFA adopts an approach that explicitly recognizes that deviations from the production frontier may arise from two distinct sources:

1. Technical inefficiency: Intrinsic shortcomings in the firm’s ability to operate on the frontier due to controllable or managerial factors.
2. Stochastic noise: Exogenous shocks, measurement errors, or other random factors beyond the firm’s control (e.g., weather events, market fluctuations).

This crucial ability to separate technical inefficiency from stochastic noise represents the main advantage of SFA over purely deterministic methods and was the reason that guided the choice of this method instead of the DEA. It allows for a more realistic estimation of inefficiency, free from random disturbances, thereby providing a more accurate and informed picture of productive performance and areas for improvement.

The core principle behind SFA lies in the specification of the production function and the composition of its error term.

The model is formulated as follows:

(1) $$Y_{it}=\;\exp\;(x_{it}^{\prime }\beta +\;V_{it}-\;U_{it})$$

Each component of the model can be interpreted as follows:

$Y_{it}$ represents the value of the livestock output achieved by firm i in period t. It is the variable to be explained.

$x_{it}$ is a vector of explanatory variables (production inputs such as capital, labor, raw materials, and other factors influencing production) for firm *i* in period *t*. These are the factors controlled by the firm that affect its productive capacity.

β is a vector of unknown coefficients quantifying the impact of each variable in $x_{it}$ on production, whose estimation is fundamental for defining the potential “frontier”.

$V_{it}$ is the component that captures the “stochastic noise” or random error. It is assumed that the $V_{it}\;$ comprises independent and identically distributed (i.i.d.) random errors following a normal distribution with zero mean and variance $\sigma_{v}^{2}$, i.e., $N(0,\;\sigma_{v}^{2})$. These errors represent factors beyond the firm’s control, such as unexpected weather variations, measurement errors in the data, or temporary economic shocks. It is important to emphasize that they are independent of $U_{it}$.

$U_{it}$ is the crucial component representing technical inefficiency in production. Unlike $V_{it}$, the $U_{it}\;$ comprises non-negative random variables. This makes sense, as a firm can operate below the frontier (positive inefficiency), but cannot produce more than what the stochastic frontier (which already includes noise) allows. The greater the value of $U_{it}$, the higher the technical inefficiency.

A key innovation in SFA, particularly evident in the context of panel data and developed by [38], is the assumption that the inefficiency effects, $U_{it}$, are not simply random variables with a fixed distribution (such as the half-normal), but may instead be influenced by firm-specific factors that vary over time. The $U_{it}$ values are obtained from the truncation (at zero) of a normal distribution with mean $z_{it}^{\prime }\delta$ and variance $\sigma^{2}$. More formally, the technical inefficiency effect $U_{it}\;$ can be specified as follows:

(2) $$U_{it}=\;z_{it}^{\prime }\delta +\;H_{it}$$

where

$z_{it}$ is a vector of explanatory variables that influence the technical inefficiency of firm *i* in period *t*. These variables may include managerial, structural, or environmental factors, or any element believed to affect the firm’s ability to operate efficiently.

δ is a vector of unknown coefficients that describe the impact of each variable in $z_{it}$ on inefficiency.

$H_{it}$ is a random variable defined by the truncation of a normal distribution with mean zero and variance $\sigma^{2}$, with truncation point −$z_{it}\delta$. This specification ensures that $U_{it}$ is non-negative, as required by inefficiency theory.

The estimation of the parameters in the SFA model is generally carried out using the Maximum Likelihood Estimation (MLE) method. This approach allows for the simultaneous estimation of both the production frontier parameters β and the inefficiency model parameters δ. The likelihood function is expressed in terms of the variance parameters $\sigma_{T}^{2\;}$=$\;{\;\sigma }_{v}^{2}$ +$\;\sigma^{2}$, and the ratio α =$\;\sigma^{2}{/\sigma }_{T}^{2}$, which indicates the proportion of the total error variance attributable to inefficiency. A value of α close to one suggests that most of the deviation from the frontier is due to technical inefficiency, whereas a value close to zero indicates that stochastic noise prevails.

Once the model parameters are estimated, the ultimate goal is to predict the technical efficiency for each firm and time period. The technical efficiency of production for firm *i* at observation *t* is defined as follows:

(3) $${TE}_{it}=exp\;({-z}_{it}\delta \;\;-H_{it})\;$$

The values of ${TE}_{it}$ range between 0 and 1, where 1 indicates full efficiency (i.e., the firm operates on the stochastic frontier), and values below 1 represent increasing degrees of inefficiency. The prediction of these efficiency scores is based on the conditional expectation of ${-U}_{it}$, given the model assumptions, thus providing an estimate of the relative efficiency of each production unit.

In the present study, the production function was specified in a log-linear form, with the logarithm of total farm revenue used as the dependent variable, while the explanatory variables (productive inputs) included in the model were the logarithms of all the considered costs. As highlighted in [38], the production function in Model (4) can be considered as the linearization (by means of logarithms) of the Cobb–Douglas production function underlying the model.

The functional form adopted is therefore the following:

(4) $${log(VoP}_{it})\;\;=\;\beta_{0}+\beta_{1}log\left(CurC_{it}\right)+\beta_{2}log\left(ESC_{it}\right)+\beta_{3}log\left(CapC_{it}\right)+V_{it}-U_{it}$$

Regarding the technical inefficiency model, in accordance with the specification proposed by [38], it was assumed that inefficiency depends on a set of structural and managerial characteristics of the firm; specifically, the inefficiency term $U_{it}$ was modelled as follows:

(5) $$U_{it}=\;\delta_{0}+\delta_{1}\left({LivU}_{it}\right)+\delta_{2}\left({UAA}_{it}\right)+\delta_{3}\left({LabU}_{it}\right)+\delta_{4}\left(TEO\_PMeat\right)+H_{it}$$

where the variables have been defined in Section 2.1.

## 3. Results and Discussions

The present section is organized by first introducing brief information and descriptive statistics regarding the characteristics of the farms in the sample and the variables used in the econometric analyses; then, econometrics results are presented.

### 3.1. Descriptive Characteristics of Italian Poultry Farms

Following the selection criteria described in the previous section, the sample was composed of 122 poultry farms, for a total of 488 observations over the period from 2019 to 2022.

Most of the units (43%) are in Eastern Italy, with 30% and 18% located, respectively, in the Southern and Central regions. More than 40% of the firms are in plain areas, and 24% and 28% are in mountainous and hilly inland areas. About half of the units were established by inheritance; rent (21%), donation (16%), and purchase (10%) are less important as a way of starting livestock businesses. As for the legal form of the units, 75% of enterprises are sole proprietorship, and 22% are simple companies. As regards the employment profile, two types of farms are represented: firms that are managed with exclusively family labor (43%), and farms with a predominance of the family labor units (48%). The livestock entrepreneur has the following main characteristics: young in 13% of firms, female in 24% of units.

The distribution of farms by classes of economic dimensions (defined by Reg CE 1248/2008) is as follows: about 40% of units are of big size (EUR 1 million or above), 26% of firms are between EUR 500 thousand and 1 million, 28% fall in the class of EUR 100–500 thousand, and the remaining units are less than EUR 100 thousand. The sector is highly concentrated and mainly characterized by large-sized organizations: the biggest firms represent almost 73% of the value of livestock output and 84% of the livestock units in the sample.

Table 1 reports some descriptive statistics of the farms in the sample. In the period from 2019 to 2022, the total value of the livestock output shows a slight decrease in the first three years, but closes the period with an increase of 17% over the average in the previous three-year period.

The number of livestock units shows annual changes and an up-and-down pattern, but the period closes with a reduction in units, compared to 2019, and compared to the average size of firms in 2019–2021.

As regards the main cost categories, current costs are very relevant and increase within the period, closing with a surge of about 25% in 2022. The long-term costs and external service costs do not affect the distribution of costs, which are highly dependent on the different types of current costs, and, on average, decrease over the period, perhaps because agricultural companies were trying to cope with increases in the current costs.

The characteristics of firms, in terms of labor, are described as follows: in the investigated population, the biggest farms represent 56% of work units and 47% of family labor units (FLU); the medium–large sized units account for 22% of LabU and 25% of FLU; and medium farms account for 18% and 24%, respectively, in terms of LabU and FLU. Indeed, livestock farms are not very labor-intensive; from the group of the smallest firms to the one with the biggest units in terms of economic dimensions, the average size of farms during the period ranges, on average, from 1.7 to 3.6 LabU, and from 1.3 to 2.0 in terms of family labor units. Furthermore, as reported in Table 1, both labor aggregates show no changes in the period from 2019–2022. In this regard, evidence suggests that the employment of automation and controlled environments in large-scale poultry farming systems results in a reduction in the overall demand for labor [39,40].

**Figure 1:**
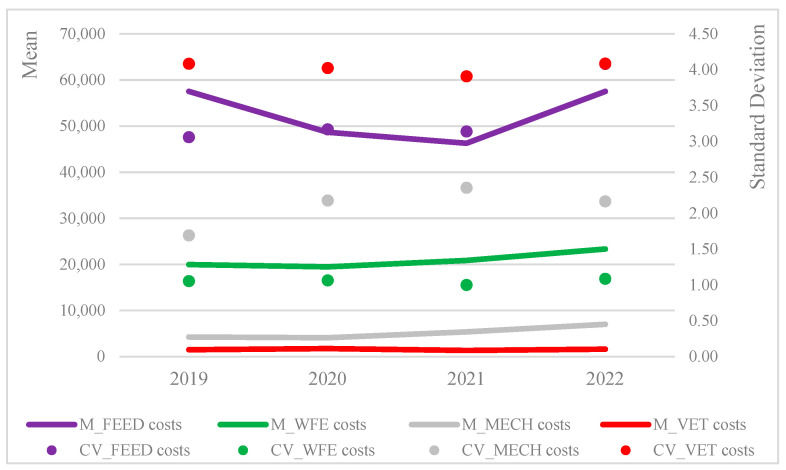
Means and standard deviations of the main cost categories (EUR).

Figure 1 reports the mean values and the standard deviations of costs by year, as well as cost categories, showing the relevance of feed costs, their increase in the last year, and a high standard deviation. The other cost categories reported slight increases in their absolute amounts, and veterinary costs show the highest variability in each year of observation. As emphasized by [41], in the observed period, the repercussions of the pandemic and the resultant protracted lockdown had substantial impacts on the livestock sector, including the poultry production system and associated value chains, nutrition, and healthcare costs.

**Figure 2:**
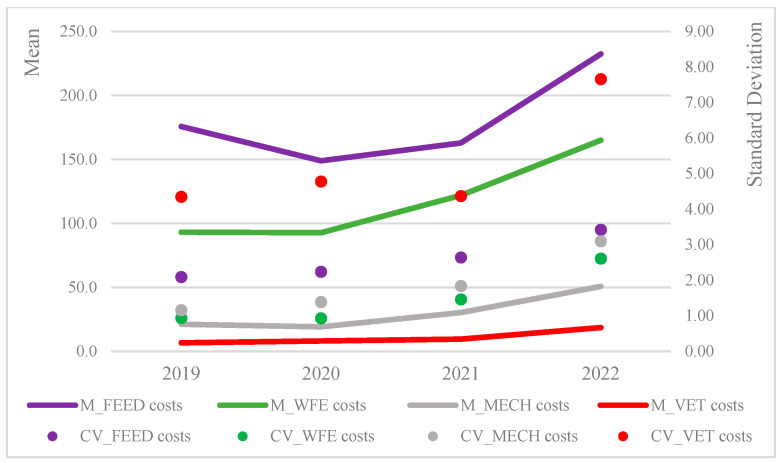
Means and standard deviations of the main cost categories by LivUs (EUR/LivU).

When costs are expressed in terms of livestock units (Figure 2), it is evident that all categories reported strong increases in the last three years, 2020–2022, especially the costs for feed and for water, fuel, and electricity. The dynamic of costs described above could reflect the impacts on Italian farms from the epidemic and pandemic events that occurred within the observed period.

The surge in chicken feed costs during the period was attributed to the escalating costs of agricultural fertilizers observed during the pandemic years, consequently exerting an indirect influence on the production costs of cereals and, by extension, of feed [42].

Poultry farming is an energy-intensive sector that requires a significant amount of fossil fuel to ensure the desired internal temperature for the health and production levels of chickens. Fuel is used for heating and cooling, lighting, ventilation, and the functioning of electric motors for the feed lines. These factors translate into high operating costs [43]. Furthermore, the increases in energy costs in 2020–2022 were exacerbated by the protracted storage times for meat- and egg-producing poultry on farms, due to forced transportation slowdowns and a sluggish market during the pandemic; the reduction in production due to epidemic outbreaks may have impacted the energy cost per unit of animal.

### 3.2. The Technical Efficiency of Livestock Farms: Results of the Econometric Analysis

Table 2 reports the parametric estimation results for SFA Models (4)–(5). The final formulation of the SFA model was decided after testing for a possible multicollinearity among the independent variables to be included; to this purpose, Variance Inflation Factors (VIFs) were calculated, yielding the following results: VIF(log(CurC)) = 1.42; VIF(log(ESC)) = 1.38; VIF(log(CapC)) = 1.05, with all values well below the limit of 5, considered in the literature as the maximum threshold.

In the first part of the model, the dependent variable, represented by the poultry farms’ VoP (expressed in logarithmic form), is explained by the three following production inputs: current costs, external service costs, and capital costs, also transformed into logarithms. The results show that both current and capital costs have positive and significant impacts on production, confirming their incisive roles in creating value. In other words, increases in current and capital costs to strengthen input endowments of farms increases the value of poultry production. The impact is particularly high regarding the current costs: with a significance at the 1‰ level and a positive sign, the coefficient of 0.643 suggests that a EUR 1 increase in current costs, ceteris paribus, would result in an increase of EUR 1.9 in the value of production. The significance of current costs is consistent with other studies, such as [17,22,25]. Capital costs are significant at 1% and exert a positive impact on the output variable: a EUR 1 increase in these costs, other inputs remaining constant, would result in a EUR 1,02 increase in the value of production. Indeed, as determined from the descriptive statistics, long-term costs have a marginal weight in the distribution of the overall cost, which is strongly determined by the current costs.

On the other hand, third-party services do not make statistically significant contributions to VoP. However, these costs are also quite low on average, as Table 1 shows. Veterinary services are essential to ensure animal welfare and as a preventive measure for disease surveillance; during the pandemic years, the provision of these services was very difficult, and costs increased [44]. Access to external services, such as veterinary services, is a particularly important issue for small farms, as they are not covered by the contractual conditions that medium and large poultry farms rely on, making them vulnerable to any crisis. The situation is different for medium and large poultry farms, as they have existing farming contracts, meaning that these services are most likely paid for by the chicken owners [5,45]. The Italian poultry supply chain is characterized by a high degree of vertical integration and contract farming [46], with processing industries and large companies frequently exerting control over all stages of production [47]. The presence of contract farming, in conjunction with forms of vertical integration within the poultry supply chain, does not reveal the costs for health and other external services, including veterinary costs [48], or the expenditures associated with health and safety services in the event of disease outbreaks or animal destruction [49]. This could be why these costs are very low and the results of the SFA analysis do not highlight a statistically significant role of external costs, due to the characteristics of the sample farms analyzed.

The second part of the model concerns the analysis of the determinants of technical efficiency based on the following explanatory variables: LabUs, LivUs, UAA, and TEO_Poultry. TEO_Poultry is coded as a dummy variable (Meat/Other). As can be seen (Table 2), all the selected variables are significantly associated with reductions in inefficiency, with negative coefficients presenting p-values of less than 5% (apart from UAA, which is significant at the 10% level); this implies that greater endowments of these factors—more labor, livestock units, and farm area—are correlated with reductions in inefficiency and, therefore, improvements in technical efficiency. The TEO_Poultry variable, which is of special interest in this study, is significant in the sense that firms specialized in poultry meat production are less inefficient (or more efficient) than the poultry firms oriented to other outputs.

Utilizing a stochastic frontier production analysis approach, Ref. [17] attained analogous outcomes by concurrently estimating TE levels and ascertaining the factors influencing the efficiency of poultry farms in Pakistan. Specifically, the findings indicated positive correlations of labor units, feed consumption, and water consumption with poultry production value; as in our study, the cost of veterinary services for animal vaccination was found to be insignificant.

The alpha value (ratio between the variance of the inefficiency model and the total variance) is 0.564; following [38], this means that about half of the random deviation in production from the frontier is due to technical (in)efficiency, so the inefficiency effects are quite significant in the analysis of the value of output of the farms. This value is quite a bit lower than the 0.98 reported in the study about poultry production in Malaysia [34], the 0.91 about broiler poultry farming in Punjab [15], and the value of 78% for the sector in Bangladesh [22], but it is greater than the 0.46 reported by [24] in South Africa. However, it should be said that the comparisons among cited studies must be carefully considered, due to differences concerning several aspects, including the model specification.

Finally, the analysis of technical efficiency shows a constant mean value of the efficiency over the period from 2019–2022, with an overall average value of 0.801 (Table 3). Based on the average of all the farms, we can conclude that, in the studied period, the observed units were producing outputs at about 80% of their potential, with the present inputs used and the current technology being employed. This means that technical inefficiency caused actual production to fall below maximum potential production by about 20%, and there is a scope for further improvements. The discussion of these results compared to the other literature is limited because of several differences in contexts, periods, and methods of investigation [28]. Limiting the comparison among studies on poultry farming by means of SFA, the values of technical efficiency obtained in this study fall within the wide range of results reported in the literature, which go from 68% in the study by [22] about the poultry broiler production in Bangladesh, to 79% in Thailand [18] and South Africa [24], to 92% in Pakistan [17], and to 95% with regard to the poultry production in Malaysia [34].

Further insights are gained by splitting the efficiency values by year and farm dimension (considering smaller farms as those below the VoP mean value, and bigger farms as those above). It is clear that bigger farms present higher mean efficiency values, maybe due to economies of scale. The difference is always quite sensible, and this conclusion is confirmed by t-tests between mean efficiency values, which yielded p<0.001 for each of the four years.

The above findings indicate that the efficiency of Italian poultry farms remained fairly constant during the period of investigation, although the period was characterized by an increase in certain costs (most of all, feed and energy costs), and that farms demonstrated resilience to external shocks. This does not mean that poultry farms have not suffered the effects of the external pandemic in the short term, but that they may have developed strategies to address these events through effective resource management. The resilience of the Italian poultry sector during the period was also observed in other countries, such as in Poland by [26]. Indeed, almost all Italian poultry production refers to the integrated supply chain, and 86% of broiler farms use multi-annual farming contracts [46]. According to the report by [46], these structural characteristics of the Italian poultry supply chain have benefits that are relevant in the context of this study: they provide greater control over the entire production process, resulting in more efficient information, innovation, and resource flows among the various components of the supply chain; greater flexibility in adapting to changes in demand; and improved supply elasticity. In addition, vertical integration and contracts help to reduce the costs of production factors within a certain range. As regards the literature, although there were some international studies dealing with the topic of contract farming and its effects on profits and costs [29], and about the higher technical efficiency of contract farmers compared to non-contract units [16,50], as regards Italy, the only study recently dealing with the topic of contract farming in the poultry sector was aimed at investigating farmers’ preferences [51]. One hypothesis, to be further tested, is that, despite the epidemic shocks, the efficiency of poultry farms remained high and constant over the period, thanks to the above sectoral characteristics. According to some reports, during the COVID period [52], as well as in recent avian influenza outbreaks [53], the poultry sector in Italy held up better than other livestock sectors thanks to the presence of a self-sufficient domestic supply chain characterized by strong vertical integration, thus not suffering from problems related to dependence on external dynamics and countries, or to other components of the supply chain.

### 3.3. Limitations and Future Research Directions

A limitation of this study is the small sample size, resulting from the application of the selection criteria based on the specialized orientation of poultry farming and the availability of data over the observed years. Nonetheless, these criteria were essential to ensuring the construction of the panel dataset and the comparability between farms based on the European standard rules set within the Farm Accountancy Data Network. Additionally, since the FADN dataset is the only source of microeconomic data based on harmonized accounting principles at the European level, future research could replicate the analyses for other EU countries, allowing for cross-country comparisons.

The analyses were based on the EU-IT FADN database that focuses on specific farm types and sizes and may not capture the full spectrum of livestock farm diversity. This could be a limitation of the sample examined, particularly considering the exclusion of small poultry farms, although the Italian poultry sector is mainly characterized by medium- and large-sized farms. A future research direction could consider the sizes of the farms and other explanatory variables to better understand the dynamics of efficiency according to the structural differences and sizes of farms. To effectively include small farms in the analyses, other data sources, such as official national business registers reporting mandatory financial statements [37], or other collection methods, such as direct surveys of representative samples of firms [20], could be used. Further variables and a different specification of the efficiency analysis model are also possible ways to further explore this study.

A further development of this research could replicate the analyses over longer time horizons than those adopted in this study, in order to follow the dynamics of efficiency performance over time and across periods characterized by different contextual conditions.

## 4. Conclusions

The aims of this study were to investigate the technical efficiency of Italian poultry farms over the period from 2019 to 2022, and to observe how efficiently they utilized their inputs with regards to controllable or managerial factors and exogenous shocks and factors beyond the firm’s control.

The findings of this study indicate that the technical inefficiency of Italian poultry farms was predominantly attributable to the management of the farms’ assets, the availability of resources and inputs, and the characteristics inherent to poultry farms. Consequently, firms’ emphasis should be directed at improving the management of capital endowment and costs, and, most of all, of current costs.

The findings of this study are corroborated by the stochastic frontier approach, which demonstrated a positive correlation between labor units and both feed consumption and production value. This result implies that the integration of management activities and resources could ensure that optimal levels of efficiency, obtained by producing maximum output from a given set of inputs, are maintained, thus reducing dependence on external factors and sensitivity to external shocks. Furthermore, the relevance of feed costs, followed by electricity, water, and fuel costs, and their sharp increases in recent years, suggest how crucial the management of these inputs is to improve the economic performance of firms and their resilience, as well as to ensure product quality and safety and animal welfare.

Recommendations for both farmers and policy makers can be drawn from the study results.

As regards farmers, recommendations for further improving farms’ economic performance in the short, as well as in the long, run are envisaged in two areas that are strictly connected. The first area regards the introduction of smart farming technologies that allow farmers to manage and monitor several aspects of poultry management. Under an economic perspective, a holistic adoption of digital technologies, such as the Internet of Things, artificial intelligence, automation, and data analytics, on one hand, improves the efficiency of use and reduces wastage of resources, reducing input costs; on the other hand, it increases productivity that, together with reductions in costs, produces higher profit margins. Given the integrated structure of the Italian poultry chain, and the diffusion of contracts between farmers and processors, digital innovation may be part of the process of innovation along the chain; furthermore, the contractual conditions, the allocation of the decision rights between the farmer and the processor, and the expected costs of the adaptation to innovations will be important aspects to investigate and monitor. A second area of recommendation, which also benefits from digital innovation, regards the systematic adoption of economic tools for the internal and continuous measurement and monitoring of farms’ efficiency performance in order to react to, prevent, and, most of all, predict events that could negatively impact firm results. The toolkit is very rich and includes financial statement and ratio analyses, as well as cost and management accounting. Constant monitoring of the economic performance of farms is particularly important to keep under control certain costs, which have high impacts on total production costs and are relevant for the technical efficiency of farms, in particular, feed and other current costs, as highlighted by the results of this study.

As regards the policy sphere, a recommendation is to implement strategies that create an integrated knowledge and innovation system for supporting the sustainability of the poultry sector. Within this system, initiatives should be integrated according to a quadruple helix approach to innovation and digitalization, aimed at creating coordination and synergies among the different intervention areas of the public policies; at fostering research, development, and technology transfer by research and education institutions; at supporting individual farms and integrated supply chains–such as with fiscal measures, subsidies, or incentives—by bearing the initial high investments costs of adopting new technologies and for increasing labor digital skills; and, finally, at informing and involving the civil society in the process of innovation.

## Figures and Tables

**Table 1:** Descriptive statistics of the main structural variables and financial items by year.

Item	2019		2020		2021		2022	
	Mean	Coeff. Variation	Mean	Coeff. Variation	Mean	Coeff. Variation	Mean	Coeff. Variation
LivU	495.1	1.9	462.6	1.9	507.8	1.9	452.4	1.9
LabU	2.6	1.3	2.5	1.2	2.5	1.1	2.5	1.1
FLU	1.7	0.5	1.7	0.5	1.7	0.5	1.7	0.5
UAA	12.4	2.0	12.6	2.0	12.7	2.0	13.2	2.1
VoP (EUR)	149,535.8	3.1	148,266.8	3.3	147,995.2	3.4	175,240.1	3.7
CurC (EUR)	138,555.6	2.2	132,070.0	2.2	139,736.4	2.2	174,113.7	2.8
ESC (EUR)	9158.6	3.2	10,252.4	3.0	9129.7	3.4	7147.8	1.9
CapC (EUR)	15,291.9	1.3	14,107.4	1.1	12,850.8	1.1	12,255.0	1.2

**Table 2:** Results of the stochastic frontier model: parameter estimation.

Item	Estimate	Std. Error	z Value	Pr (>|z|)	Signif.
(Intercept)	5.128	0.201	25.478	<0.001	***
log(CurC)	0.643	0.018	35.618	<0.001	***
log(ESC)	−0.010	0.008	−1.162	0.245	
log(CapC)	0.021	0.007	3.064	0.002	**
Z_(Intercept)	1.401	0.214	6.543	0.000	***
Z_LabU	−0.388	0.150	−2.584	0.010	**
Z_LivU	−0.001	0.001	−2.824	0.005	**
Z_UAA	−0.049	0.029	−1.709	0.087	.
Z_TEO_PMeat	−0.295	0.117	−2.510	0.012	*
sigmaSq	0.219	0.037	5.930	0.000	***
alpha	0.564	0.086	6.572	0.000	***

Signif. codes: 0: ‘***’; 0.001: ‘**’; 0.01: ‘*’; 0.05: ‘.’; 0.1: ‘ ’. Log likelihood value: −186.9423.

**Table 3:** Results of the stochastic frontier model: mean efficiency in each year by farm dimension.

Dimension	2019	2020	2021	2022	Total
<mean(VoP)	0.769	0.773	0.767	0.758	0.767
>mean(VoP)	0.941	0.937	0.950	0.957	0.946
Total	0.803	0.806	0.803	0.793	0.801

## Data Availability

The data used in the study were obtained from the RICA Agricultural Accounting Information Network, the Italian section of the EU FADN information system, at https://rica.crea.gov.it/index.php?lang=en. CREA - Research Centre for Agricultural Policies and the Bioeconomy is, on behalf of the Italian State, the liaison body with the European Commission for the implementation and management of the FADN. The raw data used in the study are available, upon request for research purposes only, at https://rica.crea.gov.it/modulo_richiesta_dati.php.

## Abbreviations

The following abbreviations are used in this manuscript:

DEA	Data Envelopment Analysis
FADN	Farm Accountancy Data Network
SFA	Stochastic Frontier Analysis
TE	Technical Efficiency
TEO	Technical–Economic Orientation
